# Strain‐Assembled Crystalline SrRuO_3_ Microtube and Emergent Curvilinear Magnetism

**DOI:** 10.1002/advs.202522085

**Published:** 2026-01-15

**Authors:** Lei Gao, Yuqian Wang, Xiangyu Lyu, Pengyu Liu, Mingtong Zhu, Jin Liu, Mengcheng Li, Ailing Ji, Qinghua Zhang, Lin Gu, Libo Ma, Zexian Cao, Nianpeng Lu

**Affiliations:** ^1^ Beijing National Laboratory For Condensed Matter Physics and Institute of Physics Chinese Academy of Science Beijing China; ^2^ School of Physical Sciences University of Chinese Academy of Sciences Beijing China; ^3^ College of Materials Science and Opto‐Electronic Technology University of Chinese Academy of Sciences Beijing China; ^4^ State Key Laboratory of New Ceramics and Fine Processing School of Materials Science and Engineering Tsinghua University Beijing China; ^5^ Leibniz Institute for Solid State and Materials Research Dresden Dresden Germany; ^6^ Songshan Lake Materials Laboratory Dongguan Guangdong China

**Keywords:** curvilinear magnetism, interfacial strain, magnetoelectronic transport, microtube, SrRuO_3_ nanomembrane

## Abstract

Strain‐induced self‐assembly presents a promising avenue for constructing novel microstructures, which can be used to simulate natural creatures and fabricate complex devices. In this work, with crystalline ferromagnetic metallic SrRuO_3_ nanomembrane as a model system, we successfully realize the fabrication of micro‐scale magnetic tubular structures. By utilizing the in‐plane anisotropic lattice strain in SrTiO_3_/SrRuO_3_ bilayer grown on SrTiO_3_ (110) substrate, we demonstrate the precise control of orientation and diameter of resulting microtubes. More interesting is that the artificially fabricated microtubes exhibit radial curvilinear magnetism due to the spin‐orbit coupling induced perpendicular magnetic anisotropy in SrRuO_3_ nanomembrane. This was confirmed by macroscopic magnetization measurement, which revealed the continuously‐rotated magnetic moment along the radial direction. Moreover, the magnetoelectronic transport measurement on a single microtube reveals that the overall magnetoresistance is closely related to the local magnetic moment distribution in the curved structure. This behavior can be modeled by integrating the magnetoresistance contributions from all longitudinal strips of the radial‐magnetized microtube. Our findings not only advance the understanding of magnetoelectric effects in curvilinear magnetism but also provide valuable insight and guidance in designing innovative spintronic devices.

## Graphical abstract

The interfacial‐strain assembly based on planar nanomembranes presents a promising approach for constructing novel magnetic microstructures. By employing the perpendicular magnetic anisotropic SrRuO_3_ freestanding nanomembrane as a model system, we exploit the interfacial strain effect within the crystalline SrTiO_3_/SrRuO_3_ bilayer, and successfully fabricate the micro‐scale tubular structures with controllable orientation and diameter. Based on this, the radial curvilinear magnetism in microtubular SrRuO_3_ was investigated.

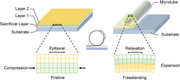

## 1 Introduction

In conventional flat lattice structures, simple ordered magnetic configurations are generally characterized by collinear spin arrangements with the Heisenberg exchange interaction, which is expressed as ‐J(*S*_i_·*S*_j_), where J is the exchange integral, and *S*_i_ and *S*_j_ are the on‐site spin states. While for the non‐collinear spin textures or curvilinear magnetism [1, 2, 3, 4, 5, 6, 7, 8, 9, 10], they are usually formed under the competitive interactions, such as magnetocrystalline anisotropy, magnetic exchange interactions, spin‐orbit coupling induced Dzyaloshinskii–Moriya interaction (DMI), etc. These curvilinear magnetism‐related spin textures include domain walls, vortices, bubbles, skyrmions, and so on. In recent years, they have attracted increasing attention for both fundamental research and potential applications, such as the next‐generation high‐density nonvolatile memories and spintronic devices.

Advances in micro‐nano fabrication have enabled the construction of complex low‐dimensional curved geometries [11, 12, 13, 14, 15, 16, 17, 18, 19, 20]. The curved surface or crystal lattice leads to space‐reversal symmetry breaking, then, combining with the spontaneously broken time‐reversal symmetry in magnetic materials, the resulting local curvature can induce many emergent physical properties, which have emerged as a novel approach to design chiral magnetic states and topological magnetism [21, 22, 23, 24, 25, 26, 27, 28, 29, 30]. Researchers have explored a wide range of magnetic configurations on curved surfaces [31, 32, 33, 34, 35, 36, 37, 38, 39, 40]. However, in most prior works, the magnetic easy axes of thin films are inclined to the in‐plane direction due to a large geometric demagnetization field, and the used materials are generally amorphous or polycrystalline. In contrast, the investigations of radial magnetic structures on high‐quality crystalline curved structures are relatively rare and necessitate exploration [37, 38, 39, 40].

Among the large number of magnetic materials, 4*d* oxide SrRuO_3_ (SRO) is a fascinating system that has attracted significant interest due to its intriguing ferromagnetic metallicity [40]. Under the spin‐orbit coupling (SOC) effect, SRO crystals exhibit strong magnetocrystalline anisotropy, with easy axis orienting along the *b*‐axis of its orthorhombic structure (Pbnm, *a* = 5.53 Å, *b* = 5.56 Å, and *c* = 7.84 Å), which corresponds to [110]_pc_ direction of the pseudo‐cube (pc) perovskite structure. Thus, the magnetic structures with different anisotropies can be formed by growing SRO films with varying crystal orientations. For instance, epitaxial SRO film grown on SrTiO_3_ (STO) (110) substrate displays magnetic moment orienting perpendicular to the film [41, 42, 43]. Then, by releasing the strain‐mismatched planar heterostructure nanomembrane, complex rolled‐up structures, such as microtubes, can be formed. Consequently, the SRO (110)_pc_ nanomembrane can be used to fabricate microstructures with divergent magnetic configurations, where the easy axes are distributed along the radial direction, distinguishing it from the previously studied curved structures with in‐plane spin orientation [31, 36]. This configuration provides a platform to investigate the curvature effect on the spin texture, and the electronic scattering mechanism can then be investigated through magneto‐electronic transport measurements.

In this work, by interfacial strain self‐assembly of the crystalline bilayer nanomembrane, we successfully fabricated the radially magnetized STO/SRO microtubular structures. Due to the lattice mismatch, strain was generated in the STO/SRO epitaxial bilayer when grown on the STO (110) substrate. Through modulating the thickness of two epitaxial oxide layers, the curvature radius of the designed microstructure with sub‐microns to tens‐of‐microns can be tuned. By leveraging the non‐equivalent in‐plane strain between the [001] and [‐110] directions of the film, the uniformly oriented microtubes were obtained. The macroscopic magnetic measurement of the microtube array indicates that the magnetic moment points along the radial direction, which manifests radial rotational symmetry of the spin configuration. Moreover, due to the strong magnetocrystalline anisotropy, the magneto‐electronic transport of a single microtube exhibits a significant anisotropic magnetoresistance effect. It has been demonstrated that, under the robust lattice‐spin coupling, the magnetoresistance of the entire microtube correlates with the local magnetic moment distribution across the curved structure, and can be effectively modeled by integrating the magnetoresistance contribution from all longitudinal strips of the microtube. The present work provides a novel pathway to fabricate microtubular magnetic structures and deepens our understanding of the electronic transport mechanism in curvilinear magnetism.

## 2 Results

### 2.1 Design Principle and Fabrication of Crystalline SrRuO_3_ Microtubes

**FIGURE 1:**
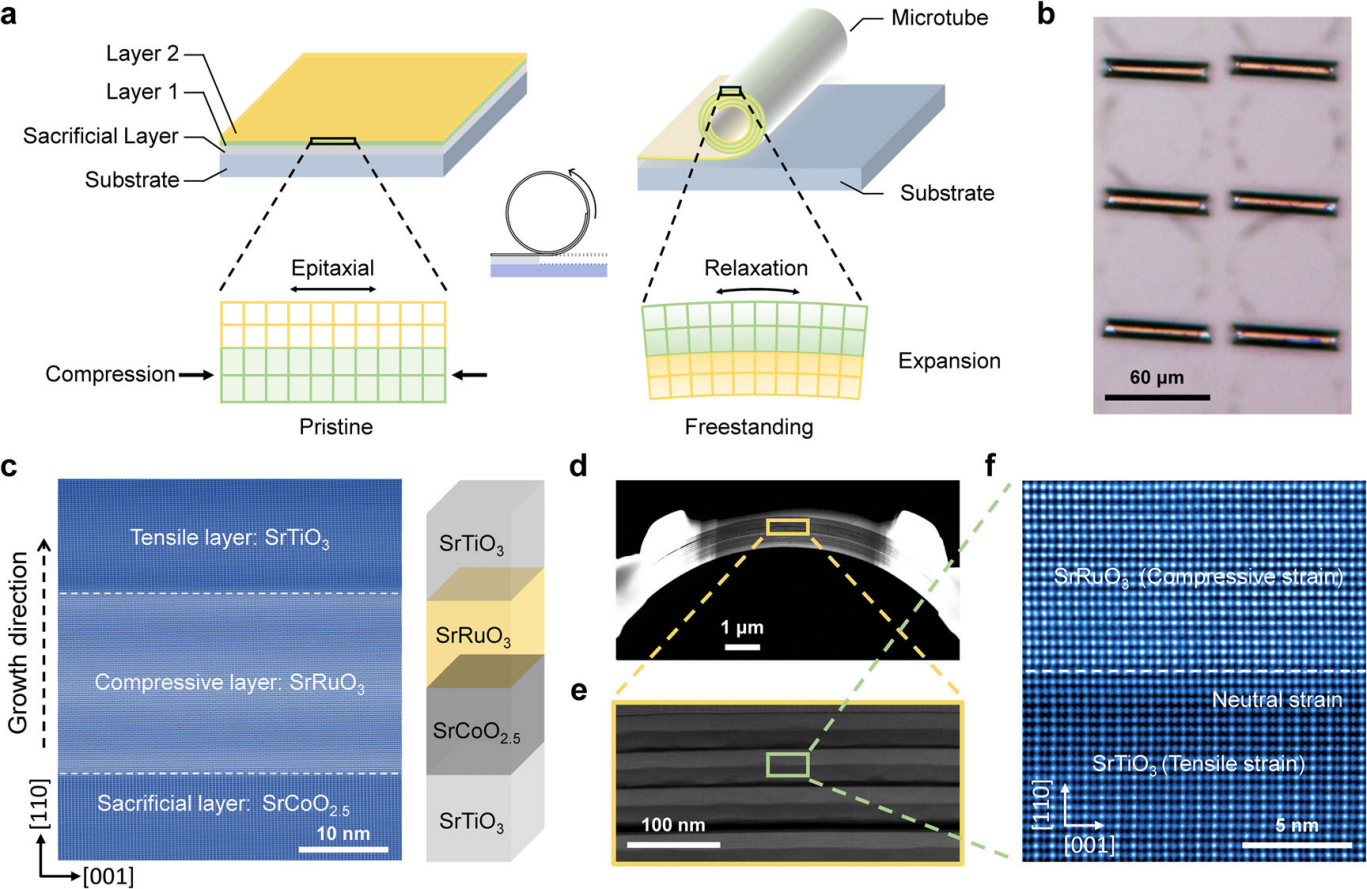
Design and fabrication of rolled‐up structures. (a) Schematic illustration of the strain‐induced self‐rolling of an epitaxial bilayer after releasing it from the substrate. (b) Optical image of rolled microtubes with a typical tube diameter of ∼10 µm. (c) Cross‐sectional STEM image and schematic diagram of the film structure grown on a STO (110) substrate. The structure consists of a sacrificial layer (SCO), a strain layer (SRO), and a component layer (STO) with each layer of ∼20 nm in thickness. (d,e) Cross‐sectional STEM images of STO/SRO microtube. (f) Zoom‐in HADDF image of the atomic‐scale bilayer interface in (e).

For the fabrication of microstructures, previously, the amorphous or polycrystalline materials were generally used for the strain assembly by ion irradiation or defect engineering [44, 45]. However, in this work, we employ the crystalline films (or membranes), from which we can precisely control the interfacial strain. The lattice mismatch between SRO (*a*_pc_ = 3.930 Å) and STO (*a* = 3.905 Å) results in intrinsic interface strain when they form the heterostructure. By introducing a 20 nm SrCoO_2.5_ (SCO) as the sacrificial layer, a crystalline STO/SRO bilayer with (001) or (110) crystalline orientation was epitaxially grown on the STO substrates (Figure S1) [46]. The alignment of asymmetric diffraction peaks between the epitaxial film and substrate in reciprocal space mapping (RSM) measurement confirms that the heterostructure films are fully strained to match the substrate lattice. After etching the SCO sacrificial layer, the pre‐strained STO/SRO bilayer rolls up into microtube structures by releasing the tensile/compressive strain in the STO/SRO bilayer, as illustrated in Figure 1a. Figure 1b shows the rolled‐up microtubes with a uniform rolling direction. Scanning transmission electron microscopy (STEM) images reveal the crystalline lattice of STO/SRO/SCO film grown on STO (110) substrate and the atomic‐scale cross‐section of the resulting STO/SRO (110) microtube (Figure 1c–f). It is seen from the heterostructure that the crystalline SRO and STO lattices are coherently grown on the SCO/STO substrate (Figure 1c). After removing the middle SCO sacrificial layer with freestanding, the SRO and STO sublayer would be compressed and stretched by each other due to the intrinsic lattice mismatch (Figure 1f). Then, under the spontaneously formed interfacial strain, the precisely controlled curved microstructures (e.g., microtube) would be formed. These findings confirm the critical role of high crystallinity of both flat heterostructure film and curved microstructure, which provides a basis for subsequent microstructure fabrication and following magnetic and electronic transport measurement.

**FIGURE 2:**
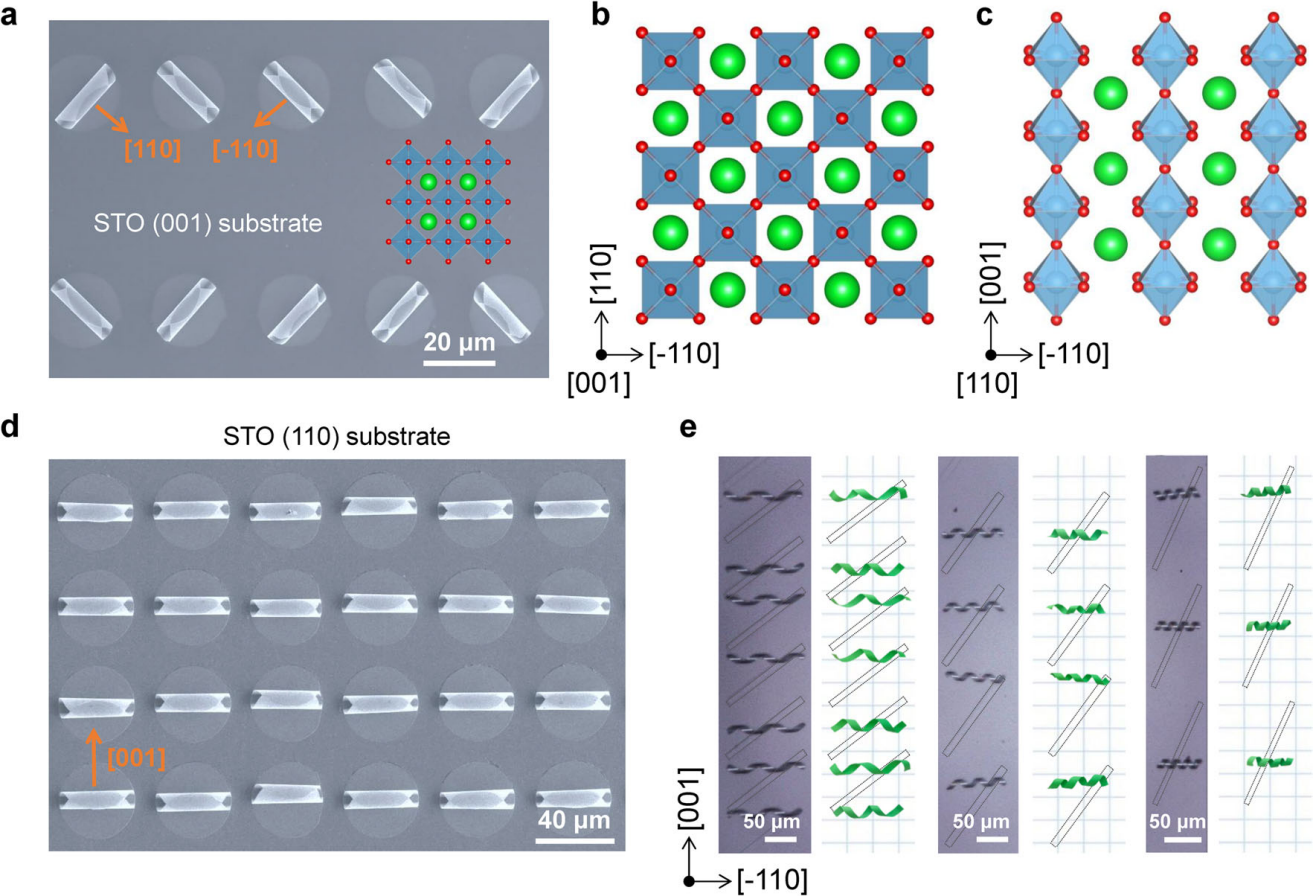
Controlled rolled‐up microstructures. (a,b) Scanning electronic microscopic image and corresponding lattice structure of rolled‐up microtubes along the two equivalent [110] and [‐110] curling direction on STO (001) substrate. (c) Side view of perovskite (110) crystal plane, from which the two non‐equivalent [001] and [‐110] axes can be noted. (d) STO/SRO coiled microtubes array with a diameter of ∼10 µm on STO (110) substrate. The curling of the microtubes is all along the [001] direction. (e) Optical microscopy images and models of helical coils formed with differently oriented strips, all of which roll up along the [001] direction.

The rolling direction of microtubes can be controlled by crystalline anisotropy of the STO/SRO nanomembranes. In our design, the rolling process occurs spontaneously along the crystal orientation by releasing the strain energy to reach an energy‐steady state. For the STO/SRO crystalline membrane grown on STO (001) substrate, the lattice strain is equivalent in [100] and [010] directions, therefore leading to the formation of two orthogonally oriented microtube structures (Figure 2a,b). In contrast, the nanomembrane grown on STO (110) substrate exhibits two non‐equivalent crystal directions, i.e., along [‐110] and [001] axes within the in‐plane orientation (Figure 2c). As shown in Figure 2d, the disc‐shaped bilayer films are all curled along the [001] crystal direction to form a uniformly aligned microtube array. In the inclined elongated strips, as demonstrated in Figure 2e, regardless of the inclinations of the strips, the bilayer STO/SRO membranes on STO (110) all preferably release internal strain by curling along the [001] direction, forming the spring‐like microstructures. The unidirectional rolling observed in the microtubes originates from the in‐plane crystalline anisotropy of the film. Specifically, the elastic constants and the bending stiffness of the crystal vary with crystallographic orientation. During the strain relaxation process, bending is likely to occur preferentially along the crystallographic direction with the smallest bending stiffness [17]. In our case, the [001] direction appears to exhibit the lowest bending stiffness, making it the energetically favorable axis for rolling.

The resultant microtube diameter is determined by crystal lattice constant (*a*_1_, *a*_2_) and thicknesses *t*_1_ and *t*_2_ of the epitaxial bilayer. The relationship can be expressed through an approximate formula $D∼{\left(t_{1}+t_{2}\right)}^{3}/3\varepsilon t_{1}t_{2}$, where *ε* represents the lattice mismatch (*a*_2_ − *a*_1_)/*a*_1_ between the two layers with the same elastic properties [14]. For the STO/SRO bilayer, *ε* is fixed at 0.77 %, and the microtube diameter can be adjusted by varying the thickness of the STO/SRO bilayer. For instance, in Figure 2a,d, the diameters of the rolled‐up single‐layer microtubes are approximately 8 and 10 µm, and the corresponding thicknesses of the STO and SRO films were chosen to be 25 and 30 nm, respectively. The rolled‐up microtubes formed by the epitaxial nanometric STO/SRO bilayer with different thicknesses are presented in Figure S2.

### 2.2 Magnetization Measurement of SRO Microtubes

**FIGURE 3:**
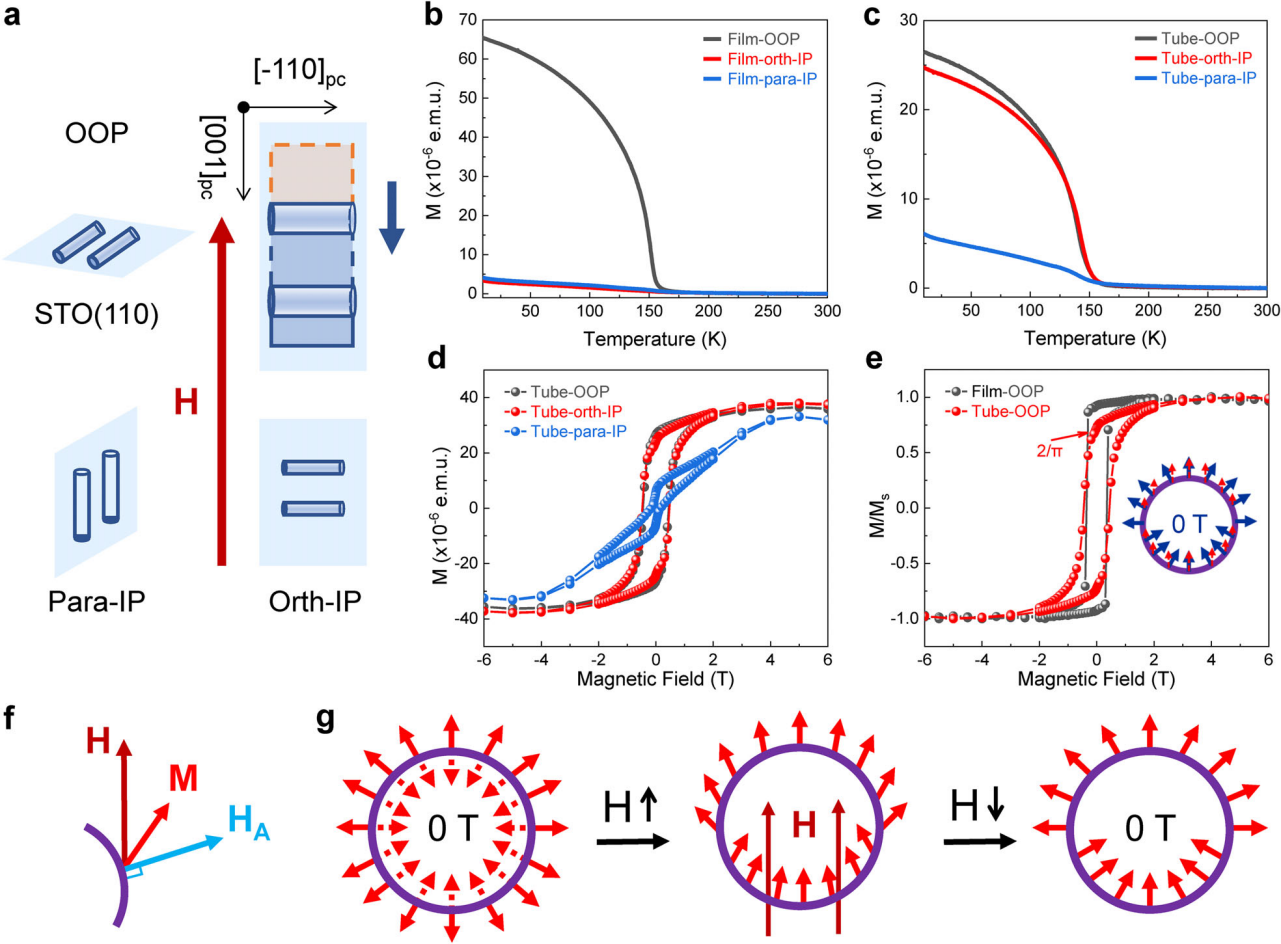
Macroscopic magnetization measurement of magnetic SRO microtubes. (a) Schematic of the three configurations in magnetic measurements. The magnetic field directions are perpendicular to the STO (110) substrate, parallel or orthogonal to the microtube array within the (110) plane, respectively. (b,c) Temperature‐dependent magnetizations of the (110) plane epitaxial film and microtube array in three configurations in (a). (d) Magnetic field‐dependent magnetization of microtube array. (e) Comparison of field dependent magnetization of the film and microtube along the out‐of‐plane direction. The inset shows the schematic magnetic moment at a strong magnetic field (red) and 0 T (blue). (f) Magnetic moment M direction in magnetic domains determined by the magnetocrystalline anisotropy field H_A_ and external magnetic field H. (g) Schematics of the spin magnetization configuration for the cross‐section of a microtube with external magnetic field dependence.

The magnetic easy axis of the SRO crystal aligns with the [110]_pc_ crystal axis due to strong magnetocrystalline anisotropy. For the epitaxial film grown on STO (110) crystal plane and the corresponding curved microtubes, there are three distinct directions, i.e., the out‐of‐plane [110]_pc_ direction of the epitaxial film (denoted as OOP), the in‐plane parallel [‐110]_pc_ direction (para‐IP), and the in‐plane orthogonal [001]_pc_ direction (orth‐IP), as illustrated in Figure 3a. The magnetic properties of the SRO epitaxial film and the microtube arrays can be revealed through magnetic measurements along these three directions.

For the planar epitaxial film, temperature‐dependent magnetization curves indicate that nearly all the remanent magnetization is perpendicular to the film, as shown in Figure 3b. The magnetic hysteresis curve of the film measured along the out‐of‐plane direction displays a rectangular hysteresis loop (Figure S3). The two in‐plane loops exhibit similar shapes with minimal remanent magnetization, suggesting the presence of hard‐axis magnetization. These results verify that the SRO epitaxial films possess uniaxial magnetocrystalline anisotropy with an out‐of‐plane easy axis and in‐plane hard axis, consistent with previous results [41, 42]. Conversely, for the microtube array (Figure 2d), the magnetic measurement along out‐of‐plane and in‐plane orthogonal directions turns out to be equivalent due to the rotational symmetry of the microtubes. The temperature‐dependent magnetization and magnetic hysteresis results (Figure 3c,d) reveal that these two directions exhibit nearly identical magnetic properties, while the measurement along the in‐plane parallel direction shows a curve like that of a planar film. The results indicate that radial magnetization moments have been formed in the interfacial‐strain self‐assembling microtubes.

Previous studies on the crystallographic and magnetic microstructure of SRO films have shown that the perpendicular magnetic anisotropy domain walls are spaced along the [001] direction with approximately several hundred nanometer intervals [47, 48]. In our study, SRO microtubes with a diameter of about 10 µm support the radially distributed magnetic domains due to the strong magnetocrystalline anisotropy. The magnetic evolution of microtubes with radial magnetic easy axis was investigated by applying an external magnetic field (Figure 3e–g). For the symmetrical circular microtube, the atomic‐scale curvature‐induced anisotropy and extrinsic DMI effect do not introduce additional tilt in magnetization of the radical magnetic structure [30], and the magnetic structure of SRO microtube is determined solely by the internal magnetocrystalline anisotropy field H_A_ that the magnetic moment of each domain should point either outward or inward, normal to the tube surface (Figure 3g).

When an external magnetic field is applied perpendicular to the longitudinal axis of the microtube, the direction of the magnetic moment in each domain is determined by both the external magnetic field H and the magnetocrystalline anisotropic field H_A_ (Figure 3f,g). As the magnetic field increases, the magnetic moment gradually tilts toward the direction of the external magnetic field. When the external field is sufficiently high, the magnetic moment at each position of the tube will align parallel to the external magnetic field. When the magnetic field is gradually reduced, the magnetic moments of each domain deflect toward the direction of the nearest neighbor's easy magnetic axis. As a result, the magnetic moments of the upper half microtube point radially outward, while those of the lower half part point radially inward. Specifically, when the external magnetic field is reduced to zero, the normalized remanence magnetization of the microtube can be calculated as $\frac{M_{0}}{M_{s}}=\frac{4\times \int_{0}^{\frac{\pi }{2}}\cos\theta \times \mathrm{rd}\theta }{2\pi r}=\frac{2}{\pi }\;∼0.637$. Coincidentally, the measured magnetic hysteresis loop shows the perfectly consistent resulting value at zero magnetic field (Figure 3e). Moreover, in contrast to the square magnetic hysteresis loop of the perpendicular magnetic anisotropy planar film, the normalized magnetic hysteresis curve of the coiled microtube array with radial curvilinear spin configuration shows a gradual decrease in magnetization during the reduction of the magnetic field (Figure 3e; Figure S3).

In curved structures, lattice distortions and local curvature variations can act as pinning sites for domain walls. The inhomogeneous strain field in a rolled‐up tube creates regions with varying magnetic anisotropy, which can trap domain walls and affect their dynamics. Figure 3e shows that rolling the film into a tube slightly increases the coercive field, as seen from a comparison of their hysteresis loops.

### 2.3 Electronic Transport Measurement of the Coiled Microtube

**FIGURE 4:**
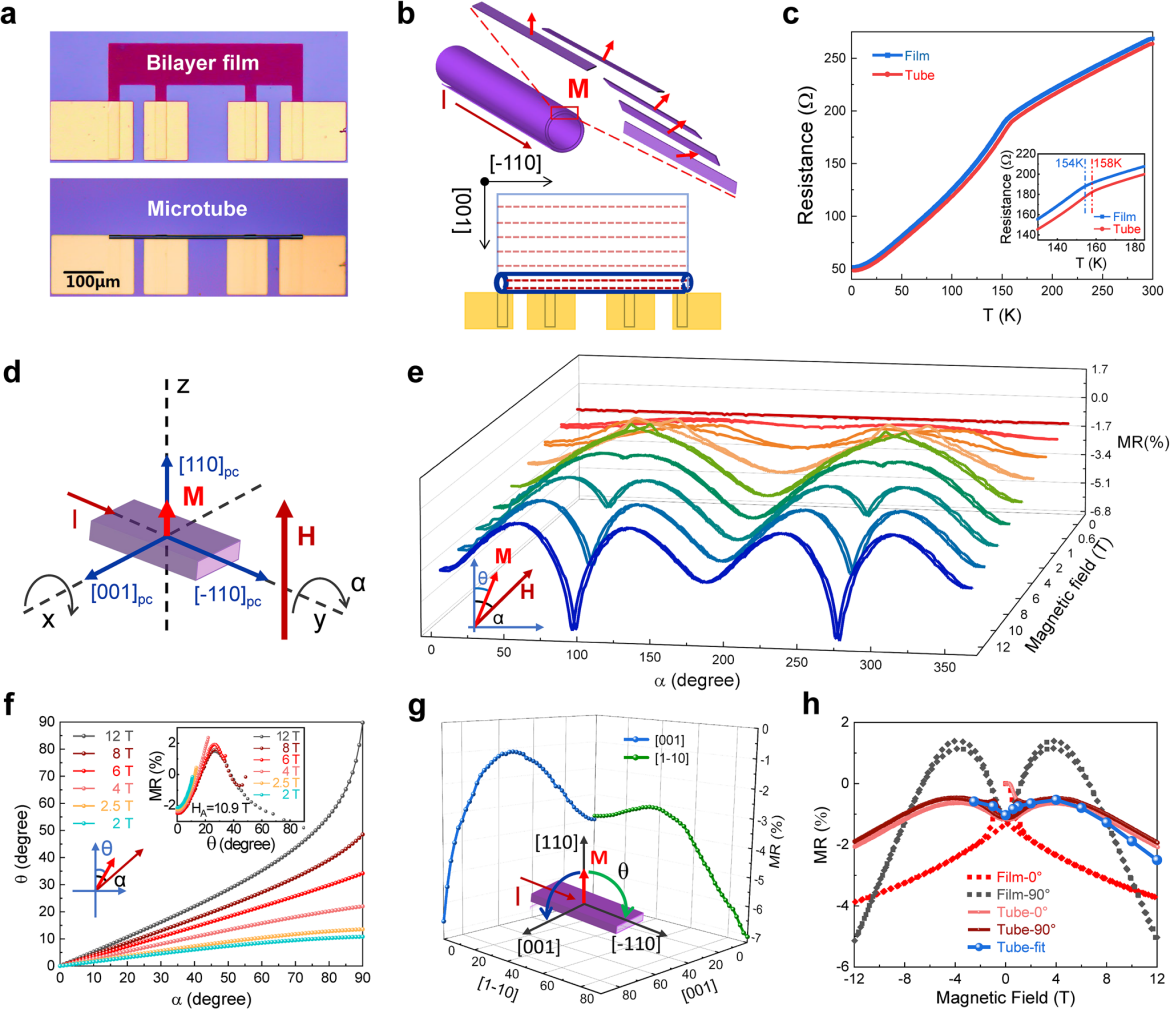
Electronic transport measurements of the film and microtube. (a) Optical images of film and microtube with four electrodes prepared by microfabrication. (b) Distribution of magnetic domain strips for film and microtube. The microtube can be regarded as composed of longitudinal strips with different magnetic moment orientations. (c) Electric resistance vs. temperature curves of film and microtube on an (110) STO substrate. (d,e) Rotation angle (α) dependent measurement configuration and corresponding magnetoresistance of the film around [‐110]_pc_ axis under different magnetic fields. Inset (e) shows the relationship between the magnetic field (H) and magnetic moment (M). (f) Relationship between the sample rotation angle α and the magnetization moment rotation angle θ under different magnetic fields. (g) Magnetic moments rotation angle (θ) dependent magnetoresistance within two crystal planes. The inset illustrates the magnetic moment rotation process. (h) Comparison of the magnetoresistance curves of the film and microtube, together with the fitting result. The angles 0° and 90°correspond to specific rotations of the sample about the [‐110] crystal axis. The 0° position is defined by the sample orientation depicted in Figure 4d.

To investigate the relationship between the electronic scattering and magnetic structure, the magneto‐electronic transport measurement (magnetoresistance) of a single microtube was conducted (Figure 4; Figures S4–S6). The planar nanomembrane and microtube samples with four electrodes are shown in Figure 4a. In the STO/SRO bilayer, the insulating nature of STO ensures that electric current flows exclusively through the conducting SRO layer. As depicted in Figure 4b, the magnetic domain strips of the microtube align with the longitudinal [‐110]_pc_ direction of SRO.

Temperature‐dependent electrical resistance measurement in zero magnetic field reveals that both the epitaxial film and microtube exhibit ferromagnetic phase transition in the range of 150–160 K (Figure 4c). The occurrence of distinct turning points indicates that the ferromagnetic transition temperature (*T*_c_) of the SRO microtube is higher than that of the strained planar film, which can be attributed to partial release of the compressive strain. This result is consistent with the previous report [49], which has shown that compressive strain decreases the ferromagnetic transition temperature of SRO films.

The initial orientation of crystalline film and magnetic field in the coordinate system is shown in Figure 4d, and the electric current (*I*) was applied along [‐110]_pc_ direction. By rotating the sample in two mutually perpendicular in‐plane directions, the magnetoresistance effect was probed. Due to the anisotropy of spin‐orbit scattering, it typically exhibits anisotropic magnetoresistance, meaning that the electrical resistance is a function of the angle between the current and magnetic moment relative to the crystal axes direction [50, 51, 52, 53]. To analyze and compare the planar STO/SRO (110)_pc_ film and microtube, the sample rotation angle‐dependent magnetoresistance was investigated by applying a magnetic field aligned with different crystal axes of SRO (Figures S4–S6). For the microtube, the electric current also flows along the [‐110]_pc_ crystal axis. During the out‐of‐plane magnetoresistance measurement, the magnetic moment at each point on the microtube rotates in the plane perpendicular to this direction. Therefore, to study the anisotropic magnetoresistance caused by magnetic moment rotation, it is necessary to test the angular‐dependent magnetoresistance by rotating the film around the [‐110]_pc_ axis.

As shown in Figure 4e, by rotating the film sample in a magnetic field, angular‐dependent magnetoresistance curves were obtained. It is important to note that under different magnetic fields, the magnetic moment rotation angle differs from that of the microtube (Figure S4). When the magnetic field is sufficiently high, the magnetic moment in the film sample is expected to fully align with the magnetic field. However, as the magnetic field decreases, the influence of magnetocrystalline anisotropy field H_A_​ becomes more prominent, causing the magnetic moment to deviate from the direction of the magnetic field. Consequently, in the low magnetic field region, the measured curves exhibit jumps around the magnetic hard axis, which are attributed to the abrupt rotation of the magnetic moments.

## 3 Discussion

The Stoner–Wohlfarth model (Equations (1), (2), (3)) is utilized to simulate the magnetic moment rotation of a single magnetic domain under varying external magnetic field [54, 55, 56]. In this research, the model assumes that the magnetization is uniform and the magnetic moment rotates within the (‐110) crystal plane in accordance with sample rotation, characterized by an angle θ in relation to the magnetic easy axis. The total energy of the system includes uniaxial magnetocrystalline anisotropy energy E_A_, Zeeman energy E_H_, and demagnetization energy E_dia_. The orientation of the magnetic moment is determined by minimizing the total energy of the system. Here, M_s_ represents the saturation magnetization, K_u_ is the magnetocrystalline uniaxial anisotropy constant, and 2K_u_/M_s_ corresponds to the effective magnetocrystalline anisotropy field H_A_.

(1) $$\begin{array}{ccc} E & & =E_{a}+E_{H}+E_{\mathrm{dia}}\\ & & =K_{u}\;\cdot \mathrm{si}n^{2}\theta -HM_{s}\cos\left(\alpha -\theta \right)+\frac{1}{2}M_{s}^{2}\cdot \mathrm{co}s^{2}\theta \; \end{array}$$

By setting $\frac{dE}{d\theta }=0,$ we obtain the following equations:

(2) $$\begin{array}{c} \frac{2K_{u}}{M_{s}}\cdot sin\left(\theta \right)\;cos\left(\theta \right)\;-H\cdot sin\left(\alpha -\theta \right)\;-M_{s}\cdot sin\left(\theta \right)\;cos\left(\theta \right)=0\;\;\; \end{array}$$

and

(3) $$\left(\frac{2K_{u}}{M_{s}}-M_{s}\right)\cdot \sin\left(\theta \right)\cos\left(\theta \right)-H\cdot \sin\left(\alpha -\theta \right)=0\;$$

Magnetic hysteresis measurements have revealed that the magnetic easy axis of STO/SRO film aligns with [110]_pc_ direction and along the *z*‐axis at the starting position (Figure 4d). It is crucial to note that as the sample rotates in a magnetic field, the magnetic moment does not exactly follow sample rotation but tends to align with the easy axis of each specific region. For anisotropic magnetoresistance, the scattering of conductive electrons is primarily influenced by the magnetic moment's orientation relative to the crystal axis. The sample rotation angle (*α*) dependent magnetoresistance measured under different magnetic fields (inset of Figure 4e) indicates the discrepancy between the sample rotation angle *α* and the magnetization moment rotation angle θ.

Moreover, the rotation angle‐dependent magnetoresistance measurements on the epitaxial film deviate from the simple cos2θ behavior typically observed in isotropic or polycrystalline ferromagnetic materials [53, 57, 58, 59]. This indicates that both the directions of current and magnetization with respect to the crystal axes play significant roles. When the anisotropic magnetoresistance curves are plotted against the magnetization rotation angle θ, the curves measured under different magnetic fields should coincide. With different values of the uniaxial anisotropy constant K_u_, for a given α and external field H, we solve Equation 3 numerically to obtain θ.  In Equations (1), (2), (3), the M_s_, as measured magnetic hysteresis, is 0.36 T (approximately 1.6 µ_B_/Ru). The α‐θ relationships can be obtained by fitting different anisotropic energy K_u_ values. By adjusting the fitting parameter K_u_, the anisotropic magnetoresistance curves measured at various fields (Figure 4e) can be made to overlap when plotted against θ, as illustrated in the inset of Figure 4f. These overlapping curves correspond to the anisotropic magnetoresistance of the SRO epitaxial film with the magnetic moment rotation angle θ. Figure 4f shows the corresponding relationship between the sample rotation angle *α* and the magnetization moment rotation angle θ for an appropriately chosen value of K_u_ across different magnetic fields.

To further explore this, we rotated the film sample around two additional crystal axes (inset of Figure 4g) by placing the sample in different orientations. This allowed us to measure the anisotropic magnetoresistance with magnetic moments rotating within three different crystal planes. The sample rotation configurations around the [001]_pc_ and [110]_pc_ axes and measurement results are shown in Figures S5 and S6, respectively. The rotation angle θ‐dependent magnetoresistance curves show local minima, corresponding to the situation where the magnetic moment is parallel to the principal crystal axes (Figure 4g). As the magnetic moment deviates from the principal crystal axis, the magnetoresistance first increases and then decreases as it approaches the other principal crystal axis direction. The magnetic moment of the SRO epitaxial film can be completely transferred from the magnetic easy axis to the vertical magnetic hard axis only at high field 12 T (Figure 4f). From these results, the magnetocrystalline anisotropy field H_A_ can be deduced as large as ∼10.9 T.

The analysis presented above demonstrates that the overall magnetoresistance of the microtube comes from the cumulative effect of all‐longitudinal strips at various angles (Figure 4b). The orientation of the magnetic moment and corresponding magnetoresistance for these longitudinal strips can be determined through the angular‐dependent magnetoresistance of the planar film under different magnetic fields (Figure 4e). Figure 4h presents the magnetoresistances of both the thin film and the rolled‐up tube under different magnetic field geometries. The angles 0° and 90° correspond to the out‐of‐plane and in‐plane perpendicular field configurations for the magnetic measurements, respectively (Figures 4d and 3a). The distinct magnetoresistance of the film in the two configurations originates from the anisotropic magnetoresistance effect associated with magnetization rotation along different crystallographic axes. In contrast, the identical magnetoresistance curves for the rolled‐up tube reflect its rotational symmetry, which averages out such crystalline anisotropy. Thus, the total magnetoresistance of the microtube can be calculated by summing the parallel resistance of all strips within a circular cross‐section. The fitting curve shows a consistent trend and result, where the magnetoresistance initially increases and then decreases (Figure 4h). The initial increase is attributed to the reason that the magnetic moment in some longitudinal strips deviates from its principal crystal axis. As the magnetic field further increases, more magnetic moments in the microtube wall are turned to align with the external field that leads to lower magnetoresistance, just like the magnetic moment rotation angle (θ) dependent magnetoresistance of films (Figure 4g).

## 4 Conclusions

In summary, magnetic microtubes were designed and fabricated by the self‐assembly of crystalline SRO/STO epitaxial heterostructures. In contrast to previous reports, here the rolling direction and tube orientation are defined by the crystal orientation as well as the substrate crystal plane. The strong magnetocrystalline anisotropy inherent in the freestanding crystalline SRO nanomembrane was utilized to obtain the radial curvilinear magnetism. In the symmetrical circular microtube, the atomic‐scale curvature‐induced anisotropy and the extrinsic DMI effect cancel each other out with respect to tilting, thus preserving the purely radial orientation of the magnetization.

The magnetization behavior and electronic transport of these tubular microstructures were characterized, revealing a continuous rotating magnetic moment configuration in the microtube structure. The magnetoresistance measured on a single microtube is found to correlate with the local magnetic moment distribution across the curved structure, which can be modeled by integrating the magnetoresistance contributions from all longitudinal strips. This study not only provides a new pathway for fabricating 3D curved magnetic microstructures but also greatly deepens our understanding of the spin‐related electronic transport mechanism, providing valuable insights for designing novel spintronic devices.

## 5 Methods

### 5.1 Thin Film and Heterostructure Growth

In this work, we epitaxially deposited STO/SRO/SCO heterostructures on STO (001) and STO (110) substrates by the pulsed laser deposition method. The SCO thin film was first grown on substrates with the krypton fluoride excimer laser (λ = 248 nm), at substrate temperature of 750°C, with an oxygen pressure of 100 mTorr and a laser fluence of 1.2 J/cm^2^. The SRO layer is deposited at a substrate temperature of 700°C with an oxygen pressure of 100 mTorr and a laser fluence of 2 J/cm^2^. The STO layer is deposited at a temperature of 650°C with an oxygen pressure of 100 mTorr and a laser fluence of 1.6 J/cm^2^. After growth, the samples were cooled down to room temperature at a rate of 10°C/min.

### 5.2 Structural Characterization

The crystal structure of films was characterized by using a high‐resolution four‐circle X‐ray diffractometer (Smartlab, Rigaku). θ‐2θ scans were performed to determine the out‐of‐plane lattice constant. X‐ray reflectivity measurements were used to estimate the thicknesses of epitaxial layers. We also conducted reciprocal space mapping measurements to determine the in‐plane lattice parameter of epitaxial films.

### 5.3 STEM Characterization

The cross‐sectional samples of film and microtube were prepared by using a dual‐beam focused ion beam integrated scanning electron microscope (Thermo‐Fisher Scientific FEI G4 CX). The STEM sample of the microtube was cut from the upper part. To ensure that the cut portion of the microtube remains bent, a Pt layer was deposited on the outer surface of the tube before cutting, and Pt was deposited on both sides of the cross‐section during the cutting process to fix and maintain the bent state. The atomic structures of the film and microtube were characterized using an ARM‐200CF (JEOL, Tokyo, Japan) transmission electron microscope equipped with double spherical aberration correctors at 200 kV.

### 5.4 Magnetization Measurements

Temperature and field‐dependent magnetic measurements were performed using a superconducting quantum interference device (SQUID) magnetometer (Quantum Design, MPMS). The temperature‐dependent magnetic measurements were performed with a heating rate of 10 K/min. Diamagnetic contributions arising from the substrate were subtracted. These magnetic hysteresis measurements were conducted at 10 K. Before magnetic testing of the coiled microtube array, cyanoacrylate glue was dropped gently on the sample surface to embed and fix the microtube array. The solidified glue can protect these microtube arrays and prevent possible contact damage during fixing the sample onto the test holder.

### 5.5 Electronic Transport Measurements

Some rectangles on the STO/SRO/SCO film with 500 ×100 µm^2^ were patterned by UV photolithography (ZML10A, ZEPTOOLS), plus Ar^+^ ion beam etching. The samples after microfabrication were annealed at 400°C in air for 1 h to eliminate the surface conductivity of the STO substrate caused by Ar^+^ ion etching. The electrical contacts were coated with Ti (5 nm) and Au (100 nm) electrodes by magnetron sputtering. By soaking the sample in acetic acid, the sacrificial layer SCO was etched, and then the coiled STO/SRO microtubes were formed. The SCO was chosen as the sacrificial layer instead of Sr_3_Al_2_O_6_ and Sr_4_Al_2_O_7_ due to compatibility concerns with our microstructure fabrication process [46, 60, 61, 62]. Specifically, Sr_3_Al_2_O_6_ and Sr_4_Al_2_O_7_ are water‐soluble and would be etched during the photoresist development step, which employs an aqueous NaOH solution. This would cause the microstructures to detach and float away. In contrast, SCO is stable in alkaline developers but can be selectively etched in acidic solutions, making it a suitable choice for our lithographic process.

By using Al wire bonding to electrically connect four electrodes of the tube structure, we can conduct electronic transport measurement of the coiled microtube. The thin‐film and coiled‐tube samples were mounted on the same holder for simultaneous measurements, ensuring identical temperature, magnetic field, and sample rotation conditions. Temperature‐dependent electrical resistance was recorded during heating at a rate of 1 K/min. For anisotropic magnetoresistance characterization, the samples were installed on a single‐axis rotating sample holder (Multifield Technology). To perform rotation tests about different crystal axes, the sample was aligned such that the desired crystal axis coincided with the rotation axis of the holder. The magneto‐electronic transport was measured by using the four‐terminal method, with imposing an alternating current of 10 µA in the SRO [‐110]_pc_ direction, and measuring voltage using the lock‐in amplifiers. The magnetoresistance measurements were taken after cooling the sample in zero field (TeslatronPT, Oxford equipment) at 2 K. For the rotation angle‐dependent magnetoresistance measurement, prior to each angular scan, the magnetic moment was put into a clearly defined initial state by setting the field to its maximum value of 12 T, where magnetic moment is supposed to nearly saturate and align with the external field. Then, the magnetic field was lowered to the testing field.

## Author Contributions

Nianpeng Lu and Lei Gao conceived and supervised the research project. Lei Gao fabricated the thin films and devices, and performed the magnetic and electronic transport measurements with help from Yuqian Wang, Xiangyu Lyu, Pengyu Liu, Mingtong Zhu, Jin Liu, Mengcheng Li, and Ailing Ji. Qinghua Zhang and Lin Gu performed the scanning transmission electron microscopy measurements. Libo Ma and Zexian Cao discussed the results. Lei Gao, Libo Ma, and Nianpeng Lu wrote the manuscript with input from all authors. All authors have discussed the results and commented on the manuscript.

## Conflicts of Interest

The authors declare no conflict of interest.

## Supporting information




**Supporting File**: advs73839‐sup‐0001‐SuppMat.pdf.

## Data Availability

The data that support the findings of this study are available from the corresponding author upon reasonable request.
