# Supplementary material for: Strain‐Assembled Crystalline SrRuO3 Microtube and Emergent Curvilinear Magnetism
Source: Adv Sci (Weinh). 2026 Jan 15;13(17):e22085. doi: 10.1002/advs.202522085 (PMC13042515; doi:10.1002/advs.202522085)
Supplement: Supplementary file 1 — Supporting File: advs73839‐sup‐0001‐SuppMat.pdf. [file ADVS-13-e22085-s001.pdf]

Supporting Information

Strain-assembled crystalline $SrRuO_3$ microtube and emergent curvilinear magnetism

Lei Gao*, Yuqian Wang, Xiangyu Lyu, Pengyu Liu, Mingtong Zhu, Jin Liu, Mengcheng Li, Ailing Ji, Qinghua Zhang, Lin Gu, Libo Ma*, Zexian Cao, Nianpeng Lu*

E-mails: gaolei00@iphy.ac.cn, l.ma@ifw-dresden.de and lunianpeng@iphy.ac.cn

Magnetoresistance of film and microtube samples

The electrical resistance of magnetic thin films derives from various electron scattering, where the magnetic fields are able to affect these scattering processes. The magnetoresistance, defined as MR=[ρ(H)-ρ(0)]/ρ(0), can be used to explore magnetic scattering-related material properties, where ρ(H) and ρ(0) represent the resistivities under a magnetic field H and zero field, respectively. In the out-of-plane direction magnetoresistance measurement of the SRO epitaxial film (film-0° in **Fig. S4a** and **S4c**), a negative magnetoresistance behavior was observed, indicating that the magnetic field suppresses the spin-wave induced scattering of conductive electrons. It is well known that the magnetic domain walls play a critical role in controlling the scattering process of the conductive electrons. The rapid decline in electric resistance during the external field can be attributed to the elimination of domain walls. When the magnetic field is swept back and forth, it does not recover the initial resistance. This is due to the fact that the sweeping field does not re-introduce as many domain walls as those in the initial zero-field-cooled sample. Furthermore, for the overall butterfly-type magnetoresistance curve, the maximum electrical resistance on both sides is attributed to the increased domain walls caused by magnetization reversal, with corresponding magnetic field representing the coercivity field of the ferromagnetic sample.

Comparing to the planar film, the curved lattice structure in the microtube may introduce many pinning effects on the domain wall due to bending effect. This has been verified by the slightly increased coercive field in the microtube compared to that in the as-grown film (**Fig. 3e**). Contrary to the pristine epitaxial film, the most significant change in the magnetoresistance curve of the microtube is that the magnetoresistance first increases and then decreases with the increase of the magnetic field (**Fig. S4b** and **S4d**). Based on the magnetic field dependent spin configuration evolution (**Fig. 3**), these distinctions can be attributed to the distributed longitudinal magnetic domain strips in the microtubes.

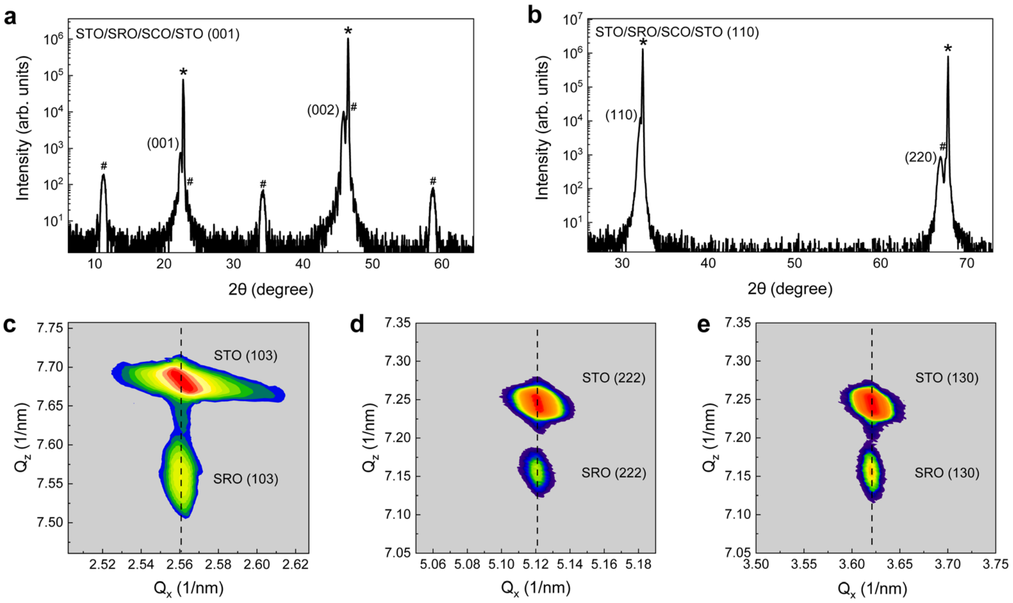

Fig. S1 Epitaxial films grown on STO substrates. a, b) XRD θ-2θ scans of STO/SRO/SCO film grown on STO (001) and (110), in which the marks (*) and (#) represent the STO substrate and sacrificial layer diffraction peaks, respectively. c-e) RSM images of STO/SRO/SCO films on STO (001) (c) and STO (110) (d, e), indicating that the in-plane lattices of these films are coherently epitaxial on the two different substrates.

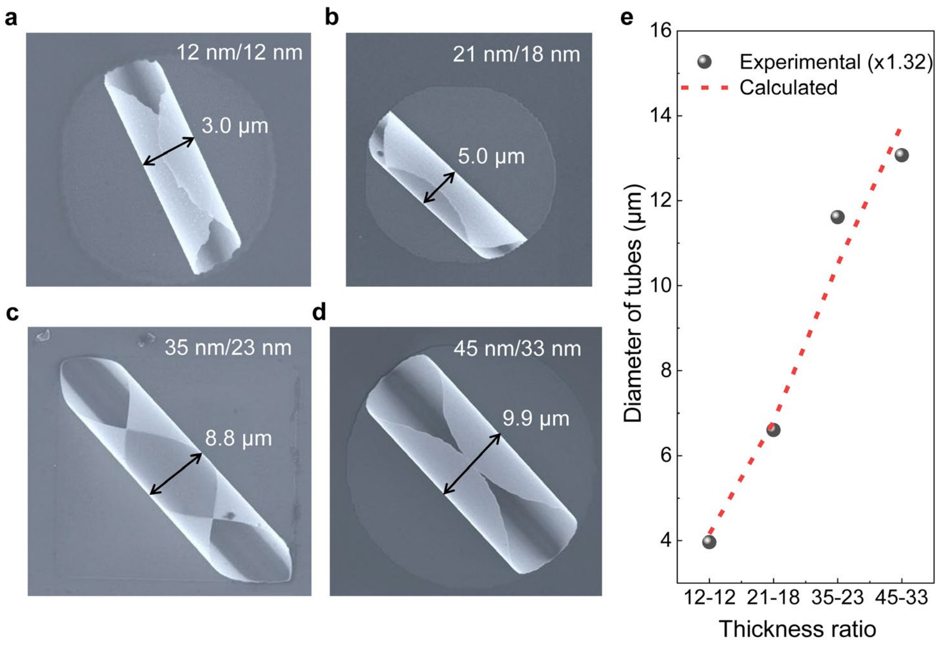

Fig. S2 Interfacial strain induced curling of the STO/SRO bilayer and formed coiled microtubes. a-d) Relationship between the microtube diameter and thickness of STO/SRO bilayers grown on STO (001) substrate. e) Experimentally observed and calculated microtube diameter which have the same variation trend, and the slight shift might be due to the error of effective film thickness.

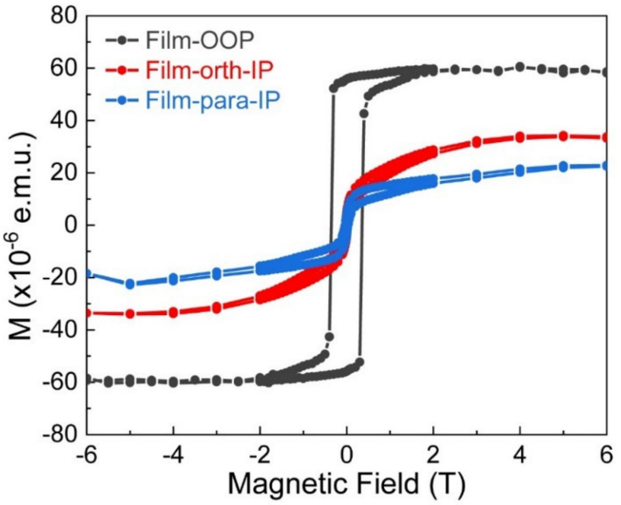

Fig. S3 Magnetic field dependent magnetization of epitaxial STO/SRO/SCO films grown on STO (110) substrate in three different configurations in Fig. 3a.

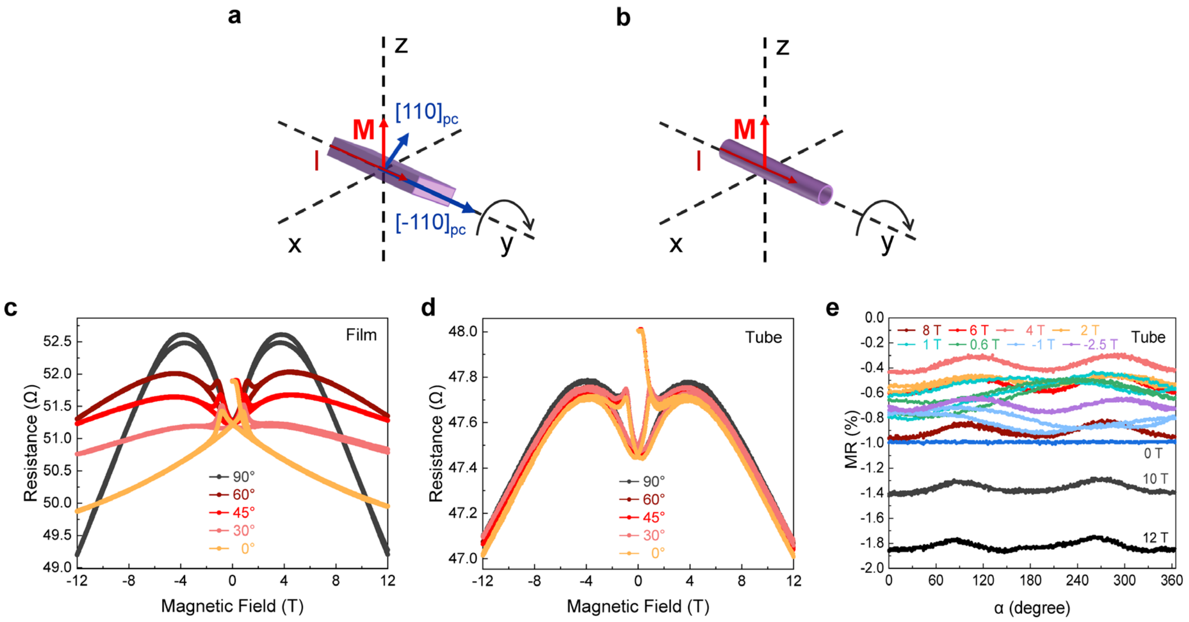

Fig. S4 Electronic transport of the STO/SRO/SCO film and STO/SRO microtube. a, b) Rotation angle dependent magnetoresistance measurement configuration around the $[-110]_{pc}$ crystal direction. c, d) Magnetic field dependent magnetoresistances of the film and microtube with magnetic field applied at different sample rotation angles. e) Rotation angle-dependent magnetoresistance of the microtube around $[-110]_{pc}$ axis under different magnetic field. Due to the rotational symmetry around this axis of the microtube, the nearly constant magnetoresistance was obtained. The slight fluctuation may be attributed to the non-uniformity of the microtube.

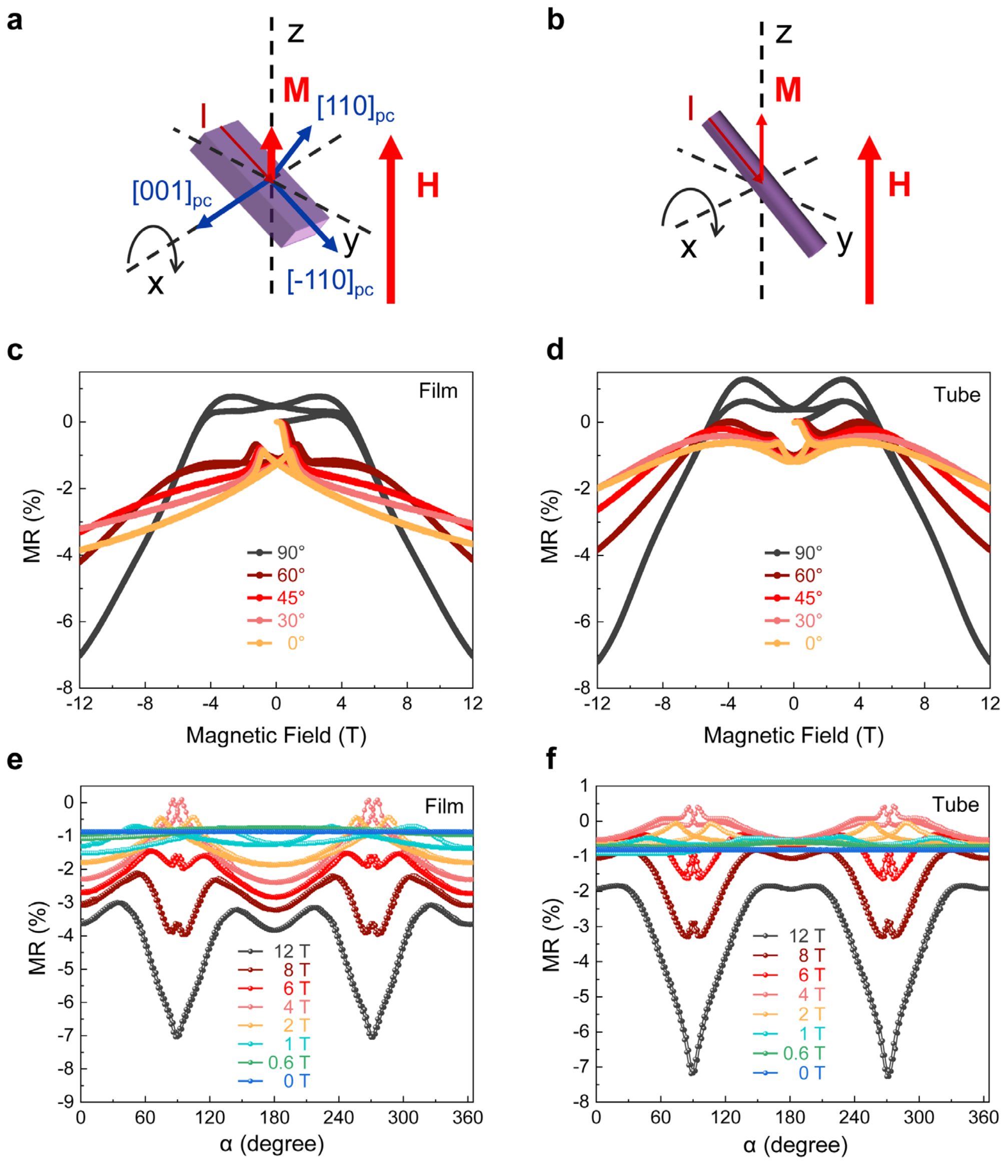

Fig. S5 Rotation angle dependent magnetoresistance measurement. a, b) Measurement configuration for the STO/SRO/SCO film on STO (110) substrate and STO/SRO microtube around the $[001]_{pc}$ crystal direction. c, d) Magnetic field dependent magnetoresistances of the film and the microtube with different sample rotation angles. e, f) Rotation angle dependent magnetoresistance of the film and microtube under different magnetic field.

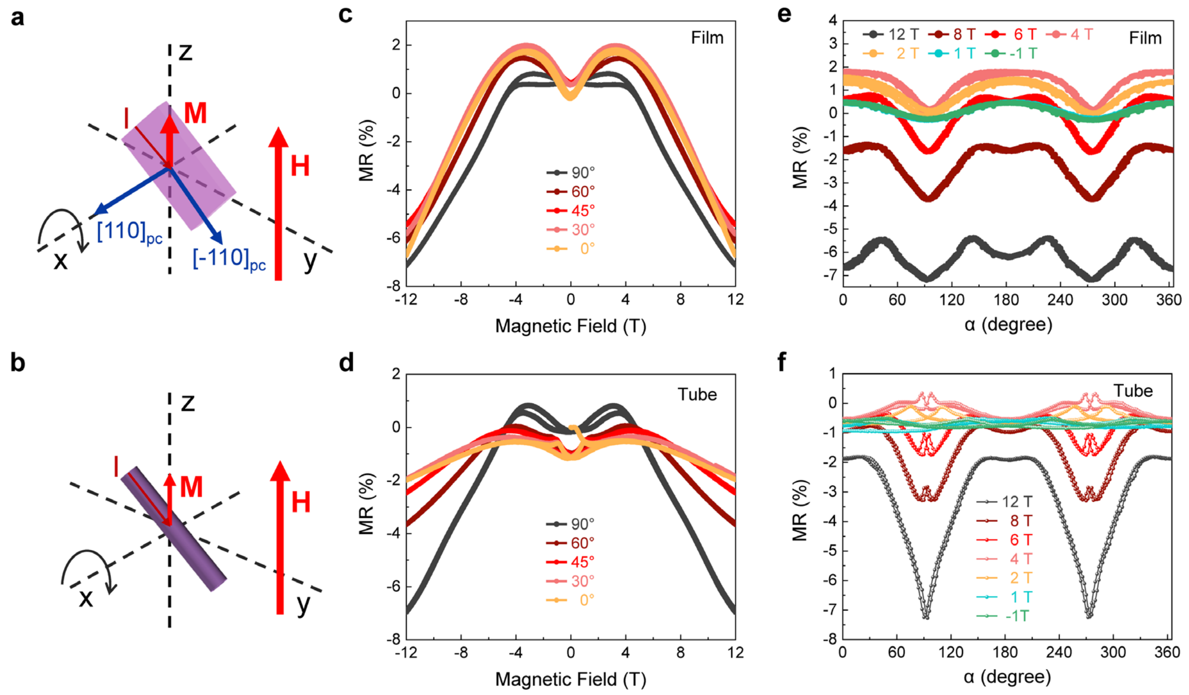

Fig. S6 Rotation angle dependent magnetoresistance measurement. a, b) Schematic magnetoresistance measurement of the STO/SRO/SCO film on STO (110) substrate and STO/SRO microtube around the $[110]_{pc}$ crystal direction. c, d) Magnetic field dependent magnetoresistances of the film and microtube at different rotation angles. e, f) Rotation angle dependent magnetoresistance of the film and microtube under different magnetic field.
